# Association of metabolic syndrome traits with urinary biomarkers in Japanese adults

**DOI:** 10.1186/s13098-021-00779-5

**Published:** 2022-01-15

**Authors:** Keiko Kabasawa, Michihiro Hosojima, Yumi Ito, Kazuo Matsushima, Junta Tanaka, Masanori Hara, Kazutoshi Nakamura, Ichiei Narita, Akihiko Saito

**Affiliations:** 1 Department of Health Promotion Medicine, Niigata University Graduate School of Medical and Dental Sciences, Niigata, Japan; 2 Division of Clinical Nephrology and Rheumatology, Kidney Research Center, Niigata University Graduate School of Medical and Dental Sciences, Niigata, Japan; 3 Department of Clinical Nutrition Science, Kidney Research Center, Niigata University Graduate School of Medical and Dental Sciences, Niigata, Japan; 4 Yukiguni Yamato City Hospital, Minamiuonuma, Niigata Japan; 5 Iwamuro Health Promotion Center, Niigata, Japan; 6 Division of Preventive Medicine, Niigata University Graduate School of Medical and Dental Sciences, Niigata, Japan; 7 Department of Applied Molecular Medicine, Kidney Research Center, Niigata University Graduate School of Medical and Dental Sciences, Niigata, Japan

**Keywords:** Albuminuria, Urinary biomarker, Chronic kidney disease, Megalin, Metabolic syndrome, Proximal renal tubule

## Abstract

**Background:**

Although metabolic syndrome traits are risk factors for chronic kidney disease, few studies have examined their association with urinary biomarkers.

**Methods:**

Urinary biomarkers, including A-megalin, C-megalin, podocalyxin, albumin, α_1_-microglobulin, β_2_-microglobulin, and *N*-acetyl-β-D-glucosaminidase, were cross-sectionally assessed in 347 individuals (52.7% men) with a urine albumin-to-creatinine ratio (ACR)  < 300 mg/g in a health checkup. Metabolic syndrome traits were adopted from the National Cholesterol Education Program (third revision) of the Adult Treatment Panel criteria modified for Asians.

**Results:**

Participants had a mean body mass index, estimated glomerular filtration rate (eGFR), and median ACR of 23.0 kg/m^2^, 74.8 mL/min/1.73 m^2^, and 7.5 mg/g, respectively. In age- and sex-adjusted logistic regression analysis, A-megalin and albumin were significantly associated with the clustering number of metabolic syndrome traits (3 or more). After further adjustment with eGFR, higher quartiles of A-megalin and albumin were each independently associated with the clustering number of metabolic syndrome traits (adjusted odds ratio for A-megalin: 1.30 per quartile, 95% CI 1.03–1.64; albumin: 1.42 per quartile, 95% CI 1.12–1.79).

**Conclusions:**

Both urinary A-megalin and albumin are associated with the clustering number of metabolic syndrome traits. Further research on urinary A-megalin is warranted to examine its role as a potential marker of kidney damage from metabolic risk factors.

**Supplementary Information:**

The online version contains supplementary material available at 10.1186/s13098-021-00779-5.

## Background

The kidneys are affected by metabolic syndrome traits, such as abdominal visceral obesity, impaired glucose metabolism, dyslipidemia, and elevated blood pressure [1–3]. The clustering of these traits has been considered to indicate a pre-disease state of lifestyle-related diseases and to favor the development of chronic kidney disease (CKD) and end-stage renal diseases [2–5]. Their clustering also leads to cardiovascular disease and all-cause death in the general population [6–8]. Although these traits are considered to be risk factors for CKD, little is known about their association with urinary biomarkers.

Urinary biomarkers can reflect pathophysiological changes in kidneys and thereby reveal links between physiological processes and diseases. The most common biomarkers for kidney damage are currently the serum creatine-based estimated glomerular filtration rate (eGFR) and proteinuria or albuminuria, which are also used to diagnose CKD [9]. A recent meta-analysis revealed that metabolic syndrome traits, with the exception of reduced high-density lipoprotein cholesterol levels, were significantly associated with albuminuria [10]. Although albuminuria is thought to be caused by morphological alterations in the glomerular filtration barrier, it might not specifically reflect pathological damage in the kidneys [11].

In addition to albuminuria and eGFR, several urinary biomarkers are focused on proximal tubular dysfunction [α_1_-microglobulin (α_1_-MG), β_2_-microglobulin (β_2_-MG), and *N*-acetyl-*β*-D-glucosaminidase (NAG)]. Although these markers are related to diabetic kidney diseases [12], little is known about their relationship with metabolic syndrome traits.

Megalin, a glycoprotein member of the low-density lipoprotein receptor family, is primarily expressed at the apical membrane of proximal tubular epithelial cells, where it plays an important role in endocytic reabsorption [13]. We previously established a sandwich enzyme-linked immunosorbent assay (ELISA) to measure the ectodomain (A-megalin) and full-length (C-megalin) forms of urinary megalin using monoclonal antibodies against the amino- and carboxy-terminals of megalin, respectively [14], and reported their clinical usefulness [15–17]. Podocalyxin (PCX), a transmembrane protein, is located at the apical membrane of glomerular podocytes and maintains the formation of intricate foot processes [18]. PCX has also been identified as a novel urinary biomarker for kidney diseases [19–21]. However, no studies have assessed the relationships of both megalin and PCX with metabolic risk factors in a general population.

In this context, the present study examined the association of urinary biomarkers with metabolic syndrome traits in a general population. Through comparison with traditional urinary biomarkers, including urinary albumin, we sought to identify potential novel urinary biomarkers that are related to the clustering of metabolic syndrome traits.

## Methods

### Study participants

This cross-sectional study was conducted by using data from a health checkup program performed as part of the Uonuma Resident Atherosclerosis Study by Anti-Sclerosis (URASA) study, Niigata, Japan. This study enrolled people who participated in a health checkup program from March 2014 to February 2016 at Yukiguni Yamato City Hospital, Niigata, Japan. Of the 352 participants, we excluded individuals with a non-fasting status (n  = 2) and those with a urine albumin-to-creatinine ratio (ACR) of 300 mg/g (33.9 mg/mmol) or more (n  = 3), leaving 347 participants as the analytic population. The study procedure was approved by the Ethics Committee of Niigata University School of Medicine (2015–1818).

### Traits of metabolic syndrome

The evaluation of metabolic syndrome performed in this study was based on the traits of metabolic syndrome listed in the National Cholesterol Education Program-Adult Treatment Panel III [22]. Accordingly, we assessed the five following components: (i) abdominal obesity, defined as a waist circumference  ≥ 90 cm in men and  ≥ 80 cm in women; (ii) fasting plasma glucose  ≥ 5.55 mmol/L or the use of any antidiabetic medications; (iii) triglyceride  ≥ 1.69 mmol/L or the use of any antihyperlipidemic medications; (iv) high-density lipoprotein cholesterol  < 1.03 mmol/L for men or  < 1.29 mmol/L for women; and (v) systolic blood pressure  ≥ 130 mmHg, diastolic blood pressure  ≥ 85 mmHg, or use of any antihypertensive medications.

Waist circumference was measured by a trained healthcare professional, with the participant in the standing maximum expiratory position. Blood pressure was measured once by a pressurized cuff at the upper arm, with the participant in a sitting position. Plasma glucose was measured using the glucose oxidase electrode method. Triglyceride and high-density lipoprotein cholesterol were measured using the enzymatic method. Abdominal visceral fat area (VFA) was measured using a bioelectrical impedance analysis device (HDS-2000 DUALSCAN; Omron Healthcare, Co., Ltd., Kyoto, Japan), with the participant in the supine position. The measurement of VFA obtained by bioelectrical impedance analysis has previously been compared with that by computed tomography, and its accuracy was confirmed in the Asian population [23].

### Urinary biomarkers

Spot urine samples were collected in the morning and were stored as 1-mL aliquots at − 80 °C on the same day. Urinary biomarkers were measured in the laboratory of Denka Co., Ltd. using stored frozen specimens. Megalin and PCX levels were measured by sandwich ELISAs as described previously [14, 20]. There are two forms of megalin in urine: the ectodomain (A-megalin) and full-length (C-megalin) forms. Briefly, we assessed A- and C-megalin by ELISA using monoclonal antibodies against the amino- and carboxyl-termini of megalin, respectively [14]. The urinary PCX level was measured by ELISA using two antibody clones that recognize the intracellular region of human PCX [20]. Urinary concentrations of albumin and creatinine were measured by turbidimetric immunoassay and the enzymatic method, respectively. α_1_-MG and β_2_-MG were measured by latex agglutination and NAG was measured by the colorimeter method. The level of each urinary biomarker was normalized to that of urinary creatinine.

### Other covariates

The general health checkups included a questionnaire, blood tests, and measurements of blood pressure, body weight, and height. The questionnaire, completed by the participants, included questions related to smoking and drinking habits, namely, whether the participant was a current smoker and whether the participant drank every day or not, respectively. Serum creatinine was measured by the enzymatic method and the eGFR was calculated as 194 × [serum creatinine (mg/dL)]^−1.094^ × (age)^−0.287^ × 0.739 (if female) [24]. Diabetes was defined as a fasting plasma glucose  ≥ 7.0 mmol/L, HbA1c  ≥ 6.5% (measured by high-performance liquid chromatography), or use of antidiabetic medication. Hypertension was defined as a systolic blood pressure  ≥ 140 mmHg, diastolic blood pressure  ≥ 90 mmHg, or use of antihypertensive medication. Body mass index (BMI) was calculated as body weight (kg) divided by height squared (m)^2^.

### Statistical analysis

Participants’ characteristics are shown as the mean [standard deviation (SD)] or percentage. We obtained Spearman’s correlation coefficients for the number of metabolic syndrome traits and abdominal VFA. The urinary biomarker trend according to the number of metabolic risk factors was tested by linear regression analysis, which included each urinary biomarker (dependent variable) as a natural logarithm because of its skewed distribution.

Logistic regression analyses were performed to estimate the association between urinary biomarkers (independent variables) and the clustering of metabolic syndrome traits (≥ 3, a dependent variable) [22]. Because there are no general standard ranges for urinary megalin and PCX, urinary biomarkers were divided by quartile into four groups in the logistic regression models. First, we ran the crude model between each urinary biomarker and dependent variable. Then, we adjusted the model by age and sex as a demographic-adjusted model. Finally, we ran the multivariable-adjusted model for the urinary biomarkers that were significantly associated with the clustering numbers of traits in the demographic-adjusted model, with adjustment by age, sex, and eGFR.

For sensitivity analyses, we estimated the association between abdominal VFA and urinary biomarkers in participants who underwent complete measurements (n  = 320). Linear regression analyzes were performed between abdominal VFA (independent variable) and each urinary biomarker (dependent variable). Crude, demographic-, and multivariable-adjusted logistic regression analyses were also performed between each urinary biomarker (independent variable) and abdominal visceral obesity (abdominal VFA  ≥ 100 cm^2^, a dependent variable) [25]. The multivariable-adjusted model for significant urinary biomarkers was used in the demographic-adjusted model, with further adjustment for eGFR.

All analyses were performed using SAS version 9.4 (SAS Institute, Inc., Cary, NC).

## Results

### Characteristics of participants according to the number of metabolic syndrome traits

The mean age and eGFR of study participants were 61.3 (SD 8.4) years old and 74.8 (SD 13.6) mL/min/1.73 m^2^, respectively. In total, 98 participants (28.2%) with clustering of metabolic syndrome traits were included. The number of metabolic syndrome traits was correlated with abdominal VFA (Spearman’s correlation coefficient  = 0.60). In terms of the descriptive characteristics other than the diagnostic traits, participants who had four or more metabolic syndrome traits were older, male, had diabetes and hypertension, and had a larger abdominal VFA, lower eGFR, and higher BMI compared with those who had a lower number of traits (Table 1).

**Table 1:** Descriptive characteristics according to the number of metabolic syndrome traits

	Number of metabolic syndrome traits				
	0	1	2	3	≥ 4
N	60	101	88	65	33
Abdominal visceral fat area, cm^2^^a^	40.8 (20.8)	50.3 (21.2)	65.1 (24.1)	86.5 (28.6)	99.8 (37.7)
eGFR, mL/min/1.73 m^2^	73.2 (11.6)	77.0 (13.9)	76.1 (12.8)	74.1 (12.4)	68.6 (18.1)
Age, years	58.1 (8.9)	59.7 (7.9)	62.3 (8.2)	64.6 (8.0)	63.2 (7.9)
Male sex, %	35.0	46.5	59.1	64.6	63.6
Body mass index, kg/m^2^	20.6 (2.1)	22.0 (2.5)	23.0 (2.9)	24.9 (2.5)	26.3 (3.0)
Waist circumference, cm	75.7 (5.8)	80.4 (7.2)	83.1 (7.8)	89.1 (6.3)	92.8 (7.4)
Current smoker, %	11.7	13.9	11.4	7.7	12.1
Drink alcohol every day, %	23.3	24.8	39.8	44.6	12.1
Systolic blood pressure, mmHg	114 (9)	123 (17)	130 (18)	133 (14)	136 (16)
Diastolic blood pressure, mmHg	69 (7)	74 (11)	78 (12)	81 (10)	83 (10)
Use of any antihypertensive medication, %	0	12.9	22.7	50.8	60.6
Fasting plasma glucose, mmol/L	5.05 (0.34)	5.25 (0.57)	5.65 (0.75)	6.06 (1.03)	6.62 (1.82)
HbA1c, %	5.6 (0.2)	5.7 (0.4)	5.8 (0.4)	6.0 (0.5)	6.4 (1.0)
Use of any antidiabetic medication, %	0	1.0	3.4	7.7	18.2
Diabetes, %	0	3.0	6.8	20.0	36.4
Hypertension, %	0	25.7	44.3	66.2	75.8

Data are shown as mean (SD) or percentage

*eGFR* estimated glomerular filtration rate

^a^The number of participants decreased by 8, 6, 8, 3, and 2, respectively

### Association between metabolic syndrome traits and urinary biomarkers

**Fig. 1:**
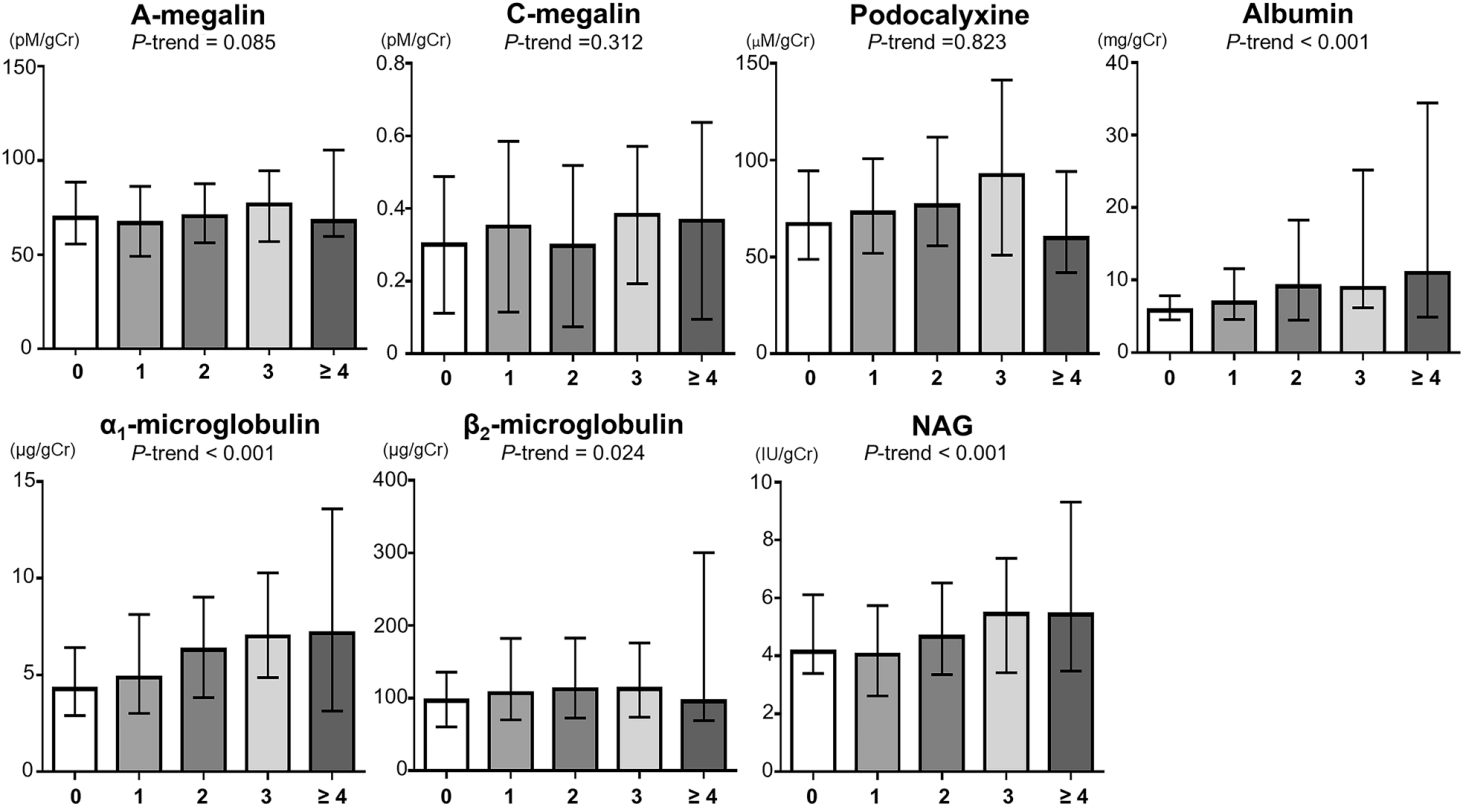
Median (interquartile range) of each urinary biomarker according to the number of metabolic syndrome traits. *NAG N*-acetyl-β-D-glucosaminidase. *P* trend values were calculated by linear regression analysis with each natural log-transformed urinary biomarker as a dependent variable

Figure 1 shows the median (interquartile range) of each urinary biomarker according to the number of metabolic syndrome traits. Participants with a higher number of metabolic syndrome traits were likely to have higher β_2_-MG (*P *trend  = 0.024) and higher albumin, α_1_-MG, and NAG (all *P *trend  < 0.001).

The crude and adjusted odds ratios of the clustering numbers of metabolic syndrome traits for each urinary biomarker are shown in Table 2. After adjustment for age and sex, A-megalin and albumin had significantly higher odds ratios of a higher clustering number of metabolic syndrome traits. After further adjustment with eGFR, these associations of A-megalin and albumin remained significant (*P* = 0.027 and *P* = 0.004, respectively) (Table 2).

**Table 2:** Logistic regression analysis between urinary biomarkers and the clustering number of metabolic syndrome traits (≥ 3)

	Crude		Demographic-adjusted		Multivariable-adjusted	
	Odds ratio (95% CI)	*P* value	Odds ratio (95% CI)	*P* value	Odds ratio (95% CI)	*P* value
Albumin	1.45 (1.16, 1.80)	< 0.001	1.43 (1.13, 1.80)	0.003	1.42 (1.12, 1.79)	0.004
A-megalin	1.20 (0.97, 1.48)	0.091	1.29 (1.03, 1.61)	0.028	1.30 (1.03, 1.64)	0.027
C-megalin	1.14 (0.93, 1.41)	0.217	1.06 (0.86, 1.32)	0.582		
Podocalyxin	1.06 (0.86, 1.30)	0.605	0.996 (0.80, 1.24)	0.975		
α_1_-microglobulin	1.38 (1.11, 1.71)	0.004	1.19 (0.94, 1.50)	0.146		
β_2_-microglobulin	1.08 (0.88, 1.34)	0.463	1.04 (0.83, 1.29)	0.761		
NAG	1.36 (1.09, 1.68)	0.006	1.18 (0.93, 1.49)	0.182		

Each urinary biomarker is treated as a quartile. The demographic-adjusted model includes age and male sex. The multivariable-adjusted model includes age (continuous), male sex, and eGFR (continuous)

*NAG N*-acetyl-*β*-D-glucosaminidase

### Association between abdominal visceral obesity and urinary biomarkers

Abdominal VFA was significantly correlated with urinary albumin, A-megalin, α_1_-MG, and NAG (all *P*  < 0.05) (Additional file 1: Table S1).

The crude and adjusted odds ratios of abdominal visceral obesity for each urinary biomarker are shown in Additional file 1: Table S2. After adjusting for age and sex, A-megalin, albumin, and NAG had significantly higher odds ratios for abdominal visceral obesity. After further adjustment with eGFR, the associations of A-megalin and albumin remained significant (*P * = 0.002 and *P*  = 0.030, respectively) (Additional file 1: Table S2).

## Discussion

This cross-sectional study examined the associations of the clustering numbers of metabolic syndrome traits with urinary biomarkers in the general adult population. We found that urinary A-megalin and albumin were each independently associated with the clustering number of metabolic syndrome traits. Corresponding results using abdominal VFA were similar to those of the clustering number of metabolic syndrome traits. Among them, the association of albumin was in line with the results of previous studies [10, 26]. However, to our knowledge, this is the first study to detect an association of the clustering numbers of metabolic syndrome traits with A-megalin, suggesting that urinary A-megalin could be a potential marker for kidney damage due to metabolic risk factors.

A number of studies have demonstrated a significant association between albuminuria and the clustering of metabolic syndrome traits. However, the pathway is not fully understood. Albuminuria has been associated with inflammation and insulin resistance, regardless of diabetes [27]. These conditions are also hallmarks of metabolic syndrome and may lead to albuminuria [28, 29]. Moreover, albuminuria has been recognized as a multifactorial marker of kidney damage, including glomerular barrier damage, tubulointerstitial alteration, and kidney endothelial dysfunction [30]. However, based on the present findings (i.e., ACR, 0–299 mg/g), these hallmarks may affect the kidneys, in particular, the tubulointerstitial condition. This is because we found a significant association of the clustering of metabolic risk traits with not only urinary albumin but also A-megalin.

Megalin is recognized as an endocytic receptor involved in the proximal tubular uptake of various proteins, including albumin, α_1_-MG, β_2_-MG, and liver-type fatty acid-binding protein [31]. It is located at the brush border, undergoes intracellular trafficking, and is shed by a mechanism that has not yet been completely elucidated. Urinary A-megalin (the ectodomain form of megalin) may reflect the recycling activity of megalin in proximal tubular epithelial cells [32] and might be increased due to endocytic metabolic loads to the cells. In contrast, urinary C-megalin or full-length megalin is correlated with the severity of diabetic kidney disease [14] and IgA nephropathy [15], which reflects established metabolic overload or damage in the endo-lysosomal systems of the cells and is excreted by exocytosis [33]. In the present study, there was a discrepancy between the results for A-megalin and other tubular markers (i.e., α_1_-MG, β_2_-MG, and NAG). Megalin is likely to be a key molecule that triggers tubular injury [34], which might increase the levels of these urinary tubular markers. Although further studies are needed to resolve this discrepancy, our findings suggest that urinary A-megalin might possibly have a different significance from other tubular markers.

Meanwhile, the present study did not show any independent association of urinary PCX with the clustering of metabolic risk traits. Urinary PCX originates mainly from the glomeruli in the kidneys [35] and is increased significantly in podocyte-injured patients (e.g., those with diabetes, kidney disease, nephrotic syndrome, and other active glomerulonephritis) [19]. Hence, our findings suggest that this “preclinical” phase of renal metabolic load associated with traits of metabolic syndrome may not involve podocytes but rather proximal tubular epithelial cells. Indeed, by examining pathological changes in the kidney, Alexander et al. found that metabolic syndrome induces renal parenchymal damages, such as tubular atrophy, interstitial fibrosis, and arterial sclerosis, but that there was no difference between groups with and without metabolic syndrome in glomerular volume [36].

Several limitations should be discussed. First, the association between each urinary biomarker and the metabolic syndrome traits was adjusted with known confounding factors, such as kidney function. However, we cannot fully exclude the residual confounding. For example, because only self-reported prescription information was available in the present study, we cannot detect who was receiving renin–angiotensin–aldosterone system inhibitors or sodium-glucose cotransporter-2 inhibitors, which could confound this association. Second, urinary biomarkers were measured only once, which might cause a misunderstanding of chronicity. Finally, because of the cross-sectional observational study design, we cannot determine temporality and causality.

## Conclusions

Our study found that both urinary A-megalin and albumin were independently associated with the clustering numbers of metabolic syndrome traits in Japanese adults. Our findings further support an indicative role for urinary A-megalin in kidney damage, which could be induced by the clustering of metabolic risk traits.

## Supplementary Information

**Additional file 1: Table S1.** Linear regression analysis between abdominal visceral fat area and urinary biomarkers. **Table S2.** Logistic regression analysis between urinary biomarkers and abdominal visceral obesity.

## Data Availability

We cannot provide individual data because we did not obtain consent from the study participants to share individual data publicly. However, the minimum dataset may be made available upon reasonable request, with ethical approval by the Ethics Committee of Niigata University.

## Abbreviations

α_1_-MG: α_1_-microglobulin
ACR: Urine albumin-to-creatinine ratio
β_2_-MG: β_2_-microglobulin
BMI: Body mass index
CKD: Chronic kidney disease
eGFR: Estimated glomerular filtration rate
ELISA: Enzyme-linked immunosorbent assays
NAG: *N*-acetyl-*β*-D-glucosaminidase
PCX: Podocalyxin
SD: Standard deviation
VFA: Visceral fat area
