# Supplementary material for: Association of metabolic syndrome traits with urinary biomarkers in Japanese adults
Source: Diabetol Metab Syndr. 2022 Jan 15;14:9. doi: 10.1186/s13098-021-00779-5 (PMC8760661; doi:10.1186/s13098-021-00779-5)
Supplement: Supplementary file 1 — Additional file 1: Table S1. Linear regression analysis between abdominal visceral fat area and urinary biomarkers. Table S2. Logistic regression analysis between urinary biomarkers and abdominal visceral obesity. [file 13098_2021_779_MOESM1_ESM.pdf]

Additional file 1

Association of metabolic syndrome traits with urinary biomarkers in Japanese adults

Table S1. Linear regression analysis between abdominal visceral fat area and urinary biomarkers

Table S2. Logistic regression analysis between urinary biomarkers and abdominal visceral obesity

Table S1. Linear regression analysis between abdominal visceral fat area and urinary biomarkers

	β (95%CI)	*P*-value
Albumin	0.003 (0.0003, 0.006)	0.034
A-megalin	0.002 (0.0007, 0.004)	0.004
C-megalin	0.004 (−0.001, 0.009)	0.166
Podocalyxyin	−0.001 (−0.004, 0.001)	0.268
α_1-microglobulin	0.004 (0.001, 0.006)	0.003
β_2-microglobulin	−0.002 (−0.005, 0.002)	0.344
NAG	0.002 (0.0002, 0.004)	0.027

Abbreviation: NAG, *N*-acetyl-β-D-glucosaminidase.

Linear regression analysis was performed with each natural log-transformed urinary biomarker as a dependent variable.

Table S2. Logistic regression analysis between urinary biomarkers and abdominal visceral obesity

	Crude		Demographic-adjusted		Multivariable-adjusted	
	Odds ratio (95% CI)	*P*-value	Odds ratio (95% CI)	*P*-value	Odds ratio (95% CI)	*P*-value
Albumin	1.34 (0.99, 1.81)	0.061	1.59 (1.16, 2.19)	0.004	1.46 (1.04, 2.06)	0.030
A-megalin	1.74 (1.26, 2.40)	<0.001	1.91 (1.34, 2.73)	<0.001	1.82 (1.26, 2.64)	0.002
C-megalin	1.25 (0.93, 1.67)	0.140	1.19 (0.87, 1.63)	0.269		
Podocalyxyin	0.86 (0.65, 1.15)	0.313	0.89 (0.65, 1.20)	0.443		
α_1-microglobulin	1.29 (0.96, 1.74)	0.093	1.25 (0.89, 1.74)	0.197		
β_2-microglobulin	0.90 (0.67, 1.21)	0.492	1.04 (0.76, 1.43)	0.796		
NAG	1.40 (1.03, 1.90)	0.032	1.56 (1.10, 2.22)	0.014	1.20 (0.81, 1.77)	0.361

Abbreviation: NAG, *N*-acetyl-β-D-glucosaminidase.

Each urinary biomarker is treated as a quartile. Abdominal visceral obesity is defined as having ≥100 cm^2 abdominal visceral fat area. The demographic-adjusted model includes age (continuous) and male sex. The multivariable-adjusted model includes age (continuous), male sex, and eGFR (continuous).
